# Measuring and modelling the quality of 40 post-disaster mental health and psychosocial support programmes

**DOI:** 10.1371/journal.pone.0193285

**Published:** 2018-02-28

**Authors:** Michel L. A. Dückers, Sigridur B. Thormar, Barbara Juen, Dean Ajdukovic, Lindy Newlove-Eriksson, Miranda Olff

**Affiliations:** 1 Impact – National knowledge and advice centre for psychosocial care concerning critical incidents, Diemen, The Netherlands; 2 NIVEL – Netherlands Institute for Health Services Research, Utrecht, The Netherlands; 3 Arq Psychotrauma Expert Group, Diemen, The Netherlands; 4 Department of Psychiatry, Academic Medical Centre, University of Amsterdam, Amsterdam, The Netherlands; 5 Department of Psychology, University of Innsbruck, Innsbruck, Austria; 6 Department of Psychology, University of Zagreb, Zagreb, Croatia; 7 CRISMART – Crisis Management Research and Training, Swedish Defence University and Royal Institute of Technology, KTH, Stockholm, Sweden; RAND Corporation, UNITED STATES

## Abstract

Disasters can have an enormous impact on the health and well-being of those affected. Internationally, governments and service providers are often challenged to address complex psychosocial problems. Ideally, the potentially broad range of support activities include a coherent, high-quality mental health and psychosocial support (MHPSS) programme. We present a theory-driven quantitative analysis of the quality of 40 MHPSS programmes, mostly implemented in European disaster settings. The objective is to measure quality domains recognized as relevant in the literature and to empirically test associations. During the EU project “Operationalizing Psychosocial Support in Crisis” (OPSIC) an evaluation survey was designed and developed for this purpose and completed by 40 MHPSS programme coordinators involved in different mass emergencies and disasters. We analysed the survey data in two steps. Firstly, we used the data to operationalize quality domains of a MHPSS programme, tested constructs and assessed their internal consistency reliability. A total of 26 out of 44 survey items clustered into three of the four domains identified within the theoretical framework: “planning and delivery system” (Cronbach’s alpha 0.82); “general evaluation criteria” (Cronbach’s alpha 0.82); and “essential psychosocial principles” (Cronbach’s alpha 0.75). “Measures and interventions applied”, theoretically a potential fourth domain, could not be confirmed to empirically cluster together. Secondly, several models with associations between domains and measures and interventions were tested and compared. The model with the best fit suggests that in MHPSS programmes with a higher planning and delivery systems score, a larger number of measures and interventions from evidence-informed guidelines are applied. In such programmes, coordinators are more positive about general evaluation criteria and the realization of essential psychosocial principles. Moreover, the analyses showed that some measures and interventions are more likely to be applied in programmes with more evolved planning and delivery systems, yet for most measures and interventions the likelihood of being applied is not linked to planning and delivery system status, nor to coordinator perceptions concerning psychosocial principles and evaluation criteria. Further research is necessary to validate and expand the findings and to learn more about success factors and obstacles for MHPSS programme implementation.

## Introduction

Communities worldwide can be confronted with disasters and crises that might have an enormous impact on the health and well-being of those affected. The health effects of disasters have received a considerable amount of attention in the scientific literature [1–10], which is helpful for public authorities and other service providers involved in the planning and delivery of mental health and psychosocial support (MHPSS) to affected populations. Internationally, disaster situations challenge governments and service providers to incorporate a potentially broad range of activities into a coherent MHPSS programme: “a community intervention that can differ in length (weeks, months, years), scope (variation in themes) and organization (number of partner organizations at different levels)”[11]. A MHPSS programme seeks to prevent, detect, mitigate, and ameliorate the often complex psychosocial problems of exposed populations. Despite the available knowledge regarding disaster-related health problems, trajectories, and risk and protective factors, strong evidence on effective MHPSS approaches is rare [12–14]. Although the structure, process and outcome of MHPSS programmes is increasingly monitored and evaluated by the organisations responsible, these evaluations often lack information that would allow comparing quality of delivery of different programmes, which could in turn inform further knowledge and quality of practice. In this article we present a theory-driven quantitative analysis of quality of 40 MHPSS programmes, mostly implemented in Europe. The objective is to measure several quality domains recognized as relevant in the literature and to empirically test associations between them, guided by the multidimensional theoretical framework presented in the following section.

### Mental health and psychosocial support programmes: Quality domains

The quality of a MHPSS programme is a multifaceted concept; bearing this in mind, Dückers and Thormar presented a framework to conceptualize the quality of MHPSS programmes based on the so-called “Donabedian model” and a categorization of “quality criteria” [11]. According to Donabedian, information about quality can be drawn from three categories: structure; process; and outcome [15]. “Structure” describes the relatively stable context in which services are delivered, including people, financial resources, tools, and equipment. “Process” denotes transactions between clients and providers throughout the service delivery system, activities, and technical and interpersonal aspects of performance. “Outcome” refers to effects on the well-being and health of individuals and populations. The three categories are not attributes of quality; they are rather classifications for the types of information that can be obtained in order to infer whether the quality of care is poor, fair, or good. Furthermore, in order to make inferences about quality, there needs to be an established relationship between the three categories [15]. The categorization of quality criteria comprises service-delivery quality criteria such as need-centeredness, safety, effectiveness, efficiency, timeliness, appropriateness, and equity [11,16–18].

In this study we use this framework as a hypothetical way of thinking about quality domains to evaluate MHPSS programmes; we empirically test associations between the domains that are described hereafter, hypothesizing that the quality domains are positively associated with one another:

- Planning and delivery system;
- Measures and interventions applied;
- Psychosocial principles;
- General evaluation criteria.

#### Planning and delivery system

The first domain is linked to the structure of a MHPSS programme which is reflected, for instance, in the availability of competent service providers (professionals, trained volunteers), trauma experts, government officials, and representatives from the local affected community. According to international guidelines, a multi-agency MHPSS planning group should exist before a disaster strikes. Moreover, the programme requires good coordination/management, sufficient funding, and should be based on evidence-informed guidelines (integrated in disaster plans that are regularly updated, tested, and facilitated). Components like these are the building blocks of what is called a “planning and delivery system”, the coordinating centre of the MHPSS programme [19,20].

#### Measures and interventions applied

Programme actions are embedded in a process that ideally is responsive to the needs and problems of affected people. Here, we conceive measures and interventions plotted along the lines of a stepped model of care, including basic psychological first aid and community activation as well as more focused support and professional mental health services appropriate to the needs of the affected. Needs assessments, dissemination of information leaflets, site visits, initiatives to strengthen social support and participation, and commemoration ceremonies are some of the recommended interventions. Furthermore, for people with health complaints or symptoms (e.g. post-traumatic stress disorder), the provision of evidence-based psychotherapy approaches such as trauma-focused cognitive behavioural therapy or eye movement desensitization and reprocessing (Schnyder et al.; Kazlauskas et al.; Schnyder et al.; for other examples of measures and interventions, see Te Brake et al.; Bisson et al.; Suzuki et al.; Witteveen et al.) [12,19,21–25] is highly recommended.

#### Psychosocial principles

When it comes to outcome, one can think of changes in the well-being, health and functioning of target groups in the short and longer term. It is challenging to ascribe these health-related aspects to specific events and circumstances, particular measures and interventions, or to something less tangible like a planning and delivery system. In the case of MHPSS programmes, besides anticipated positive outcomes of ideal intervention—of which the realization is difficult to test (e.g. reduction of stress-related health problems such as post-traumatic stress disorder or depression and improvement in well-being)—there are other objectives that are considered explicitly essential. Hobfoll and colleagues [13] identified five aspects they claim crucial to embed in strategies to promote health and wellbeing after disasters, based on a synthesis of available scientific evidence. Measures and interventions should promote: a sense of safety; calming; self- and community efficacy; social connectedness; and hope [12,13]. Currently these essential principles have been embedded in different evidence-based guidelines [12,25,26]. We therefore consider these principles as potentially suitable outcome indicators for a MHPSS programme.

#### General evaluation criteria

General quality criteria for health service delivery constitutes a fourth domain. In the past decades, several quality features have been distinguished in the international health sciences literature [16–18]:

- Need-centeredness: provide services that are respectful of and responsive to preferences, needs, and values of affected people, ensuring that their values guide all decisions;
- Safety: avoid harm to people from services that are intended to help them;
- Effectiveness: provide services based on scientific knowledge to all who could benefit from them, and refrain from providing services to those unlikely to benefit, thus avoiding both underuse and overuse, respectively;
- Efficiency: avoid waste, including waste of equipment, ideas, and energy;
- Timeliness: reduce waiting and sometimes harmful delays for those who receive and those who provide services;
- Equity: provide services without variation in quality due to personal characteristics, such as sex, ethnicity, religion, geographic location, and socioeconomic status [11].

## Methods

### Survey

In order to analyse and compare the quality of MHPSS programmes we conducted a survey in which information was collected on each of the four domains. Validated evaluation tools, covering the content of evidence-based guidelines and elements from all four domains are scarce. In this study we used data collected during the EU project “Operationalizing Psychosocial Support in Crisis” (OPSIC). In the OPSIC project an online survey tool was developed for this purpose based on interviews and a systematic assessment of existing MHPSS guidelines and handbooks, guided by the domains described in the previous section [27]. The instrument was filled out by programme coordinators of MHPSS programmes implemented in reaction to calamities in different countries. The instrument contained an extensive and diverse set of queries, divided over the following sections–for a complete version of the instrument see S1 File (Thormar & Olff) or the report by Juen et al. [27]:

- Participant characteristics: function; organization; and role of organization in disaster management;
- Event characteristics: year; location (country, city/area); nature of the event; short description of event and impact; number of casualties and survivors (including level of injury); and estimation of other losses (property, livelihood, livestock);
- Organizations involved in provision of MHPSS;
- MHPSS target groups/beneficiaries;
- MHPSS interventions provided during preparation, response, and recovery phases (e.g. planning and delivery system, funding, training, supervision, dissemination of information, assessments and monitoring, community activities, long-term coordination, support for staff and volunteers);
- Essential psychosocial principles: importance and level of success;
- Evaluation: general evaluation criteria and “good”/“bad” practices.

In line with the grant agreement of the OPSIC project, all decisions, planned activities, methods and instruments, as well as the progress in their application were discussed with consortium partners including an ethical advisor, both in the planning and execution phases of the project. Moreover, the entire OPSIC project was formally reviewed periodically by an ethical advisory board comprised of international experts. With regard to the particular survey study and analysis described here, neither the advisor nor the ethical advisory board deemed a formal review by a medical ethical committee or an institutional body relevant or necessary; it is not a clinical study and thus does not fit criteria for review. No patients or disasters victims were involved. Participation by programme coordinators was voluntary and under the condition that results would only be presented at an aggregated and anonymized level. Examples are presented without country and location characteristics and event details are described in general terms.

The invitation to participate was disseminated to programme coordinators via the OPSIC consortium and the network of the Reference Centre for Psychosocial Support of the International Federation of Red Cross and Red Crescent Societies. 40 programme coordinators participated in the survey between January 26 and April 22, 2014.

### Operationalization

The survey data was used to empirically operationalize the quality domains of MHPSS programmes (see S2 File). A total of 44 items were preselected from the survey (listed in Table 1). The first domain included recommended elements of a planning and delivery system and the preferable involvement of key actors [12,19,20,24,25] (10 survey items). For the second domain, survey items on measures and interventions were selected from international guidelines [12,24,25,27,28] (14 survey items). For each item in these first two domains, a 1 was assigned when it was present, a 0 when absent. The five essential psychosocial principles—promoting a sense of safety, calming, self- and community efficacy, social connectedness, and hope [12,13,25,26]—both in terms of perceived importance and self-reported level of success, were included in the third domain (“within the [measures and] interventions for beneficiaries, how important were the essential [principles] (…) and to which degree do you think you succeeded in reaching the aim”; 10 survey items). These items were measured on a scale from 0 to 5 (importance and success level ranging between “not …” to “very …”). We labelled the fourth domain general evaluation criteria and included survey items covering service evaluation criteria, such as programmes’ level of need-centeredness, safety, effectiveness, efficiency, timeliness, appropriateness, and equity [11] (10 survey items), all measured on a scale from 0 to 10 (ranging between “not very …” to “very …”).

**Table 1:** MHPSS programme quality items per domain and distributional information per item.

Item		Mean	N	IQR	Min-Max
	***Planning and delivery system***				
PD_1	Multi-agency planning group	0.49	35	1	0–1
PD_2	Politicians or government officials involved in planning group	0.76	38	0	0–1
PD_3	Local individuals involved in planning	0.77	39	0	0–1
PD_4	Trauma experts involved in planning group	0.78	36	0	0–1
PD_5	Good cooperation with other actors	0.69	35	1	0–1
PD_6	Psychosocial care plan to use in emergencies	0.75	40	.5	0–1
PD_7	Overall emergency plan	0.63	40	1	0–1
PD_8	Build upon existing guidelines	0.54	39	1	0–1
PD_9	Existing psychosocial services fully mapped	0.67	36	1	0–1
PD_10	Psychosocial care plan tested through exercises	0.46	39	1	0–1
	***Measures and interventions applied***				
MI_1	Mental health complaints assessment	0.36	36	1	0–1
MI_2	Integrated co-ordination point for long-term	0.47	36	1	0–1
MI_3	Appropriate conditions/facilities for communal, cultural, spiritual and religious healing practices	0.76	34	0	0–1
MI_4	Needs of minority or particular vulnerable groups taken into account	0.70	37	1	0–1
MI_5	Site visits	0.58	31	1	0–1
MI_6	Legal advice	0.56	36	1	0–1
MI_7	Financial assistance	0.67	36	1	0–1
MI_8	Stepped model of care	0.77	31	0	0–1
MI_9	Professional treatment for acute stress or referral	0.78	37	0	0–1
MI_10	Memorial services	0.57	30	1	0–1
MI_11	Information meeting with the affected	0.78	32	0	0–1
MI_12	Telephone helpline	0.55	31	1	0–1
MI_13	Psychoeducational leaflets	0.75	36	0.5	0–1
MI_14	Co-ordination centre for aftercare	0.43	30	1	0–1
	***Essential psychosocial principles***				
EP_1	Successful in providing safety	4.06	32	1	0–5
EP_2	Successful in promoting connectedness	3.84	32	1	1–5
EP_3	Successful in promoting a sense of calmness	3.74	35	1	1–5
EP_4	Successful in promoting self and community efficacy	3.50	34	1	2–5
EP_5	Successful in igniting hope	3.26	34	1	0–5
EP_6	Importance of providing safety	4.55	38	0	0–5
EP_7	Importance of promoting connectedness	4.68	38	0	3–5
EP_8	Importance of promoting a sense of calmness	4.78	40	0	3–5
EP_9	Importance of promoting self and community efficacy	4.38	39	1	2–5
EP_10	Importance of igniting hope	4.44	39	1	2–5
	***General evaluation criteria***				
GE_1	Responsive to needs and problems	8.34	38	1	6–10
GE_2	Overall preparedness plan helped to respond	7.30	37	2	0–10
GE_3	Effective in addressing needs and problems acute phase	7.49	37	2	0–10
GE_4	Effective in addressing needs and problems recovery phase	7.34	35	3	0–10
GE_5	Efficient (invested resources in relation to people assisted)	8.11	35	3	4–10
GE_6	Efficient in reaching vulnerable groups	7.08	37	2	0–10
GE_7	Appropriateness given circumstances	8.54	37	1	1–10
GE_8	Contribute to safety affected people	7.91	35	2	3–10
GE_9	Contribute to safety services providers/staff	8.38	32	3	4–10
GE_10	Affected people treated equally	9.08	37	1	0–10

*Note*. N = Number of responses, IQR = Inter-quartile range, Min-Max = Minimum-Maximum.

### Analysis

The survey data were analysed in two steps. In step 1 we used the data to operationalize the quality domains of a MHPSS programme, tested constructs and assessed their internal consistency reliability. This step was needed to reduce the number of variables and to cluster variables into verified constructs to include in step 2, where associations between these constructs were examined in different models.

#### Step 1. Testing of constructs

As a first step a confirmatory factor analysis was conducted to test whether or not the items load on latent constructs corresponding with the four hypothetical quality domains. We tested whether the 44 items clustered along the four domains. Items were selected matching the respective central theme per domain (as shown in Table 1). Additional analyses were guided by the test results, combined with an assessment of internal consistency reliability to verify potential improvements. Internal consistency reliability was assessed using Cronbach’s alpha coefficient. A coefficient of 0.70 or higher was considered sufficient.

#### Step 2. Modelling of associations

As indicated, we empirically tested associations between the quality domains, hypothesizing that the quality domains are positively associated with each other. Given the relatively modest sample size, four or less variables are a desirable starting point for the statistical modelling. Step 1 was needed to determine whether it is possible to reduce the number of variables from 44 items to a substantially smaller number of theory-based domains. A lower indicator-to-sample size ratio is one advantage of working with an average domain score (or “parcel”), as opposed to including all items of a construct [29]. N.B. Little and colleagues described other advantages of working with a parcel when it comes to psychometric properties, model estimation and fit characteristics (e.g. higher reliability; greater communality; higher ratio of common-to-unique factor variance; lower likelihood of distributional variations; more, tighter, and more-equal intervals; fewer parameter estimates; lower likelihood of correlated residuals; reduced sources of sampling error) [29].

**Fig 1:**
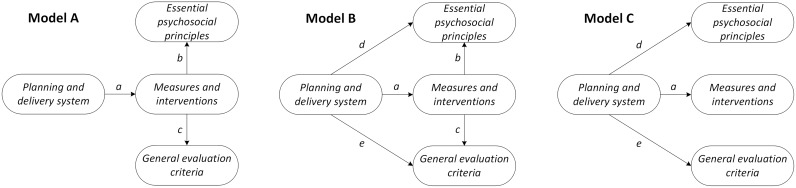
Three test models. The three test models display associations between the quality domains of a programme. In model A a more developed planning and delivery system is accompanied by a higher score on measures and interventions applied (*relation a*), resulting in higher perceived scores assigned to essential psychosocial principles (*relation b*) and general evaluation criteria (*relation c*). Model B follows the same line of reasoning but now the planning and delivery system status directly influences perceived essential psychosocial principles (*relation d*) and general evaluation criteria scores as well (*relation e*). Model C is restricted to the influence of a change in planning and delivery score on the other domains.

Fig 1 gives an overview of three test models showing possible associations between the quality domains. Model A assumes a positive association between planning and delivery system score and measures and interventions applied (*relation a*), and between measures and interventions applied and essential psychosocial principles (*relation b*) and general evaluation criteria scores (*relation c*). Model B also considers the direct effects of a more developed planning and delivery system on the other quality domain scores (*relations d and e*). In the final model we tested the influence of a change in planning and delivery score on the other three domains (*relation a*, *d and e*).

#### Modelling

During step 1 and 2 multiple models were tested using structural equation modelling (SEM) and generalized structural equation modelling (GSEM). Where SEM is suitable when variables are continuous, GSEM is applicable when working with binary and continuous variables [30]. The planning and delivery system items and the measures and interventions applied were measured on a binary scale, the essential psychosocial principles and the general evaluation criteria, continuous. GSEM was therefore applied during step 1. SEM was used in step 2 where step 1 justified calculating continuous construct scores. SEM and GSEM allow a comparison of different models based on common information criteria: Akaike’s information criterion (AIC) and Bayesian information criterion (BIC). The AIC and BIC are comparative measures of fit and so they are meaningful only when different models are estimated. Lower values indicate a better fit and so the model with the lowest AIC and BIC is the best fitting model [31]. Unlike GSEM, SEM allows computation of common model fit measures such as chi-square, Confirmatory Fit Index (CFI) and Tucker-Lewis Index (TLI), Root Mean Square Error of Approximation (RMSEA) and P of close fit (PCLOSE). The chi-square measure of fit should not be significant. CFI and TLI can range between 0 and 1. Values below .90 indicate that the model can be improved, values between .90 and.95 are acceptable, and values above .95 are good [32]. Good models, moreover, have an RMSEA value of equal to or lower than .05, values between .05 and .08 are considered acceptable, values higher than .10 indicate a poor fit [33,34]. The RMSEA is preferably close to zero with PCLOSE higher than .05 indicating a “close” fit of the model. Modelling results were interpreted based on these rules of thumb. Estimation method was maximum likelihood. All analyses were performed in Stata version 13 (StataCorp LP).

## Results

### Survey participants

The majority of the 40 programme coordinators participating in the survey were linked to the Red Cross and Red Crescent organization (75%). Asked about their function in their organization, the respondents assigned themselves to the following pre-defined categories: management (30%), programme manager (25%), volunteer (25%), desk officer (15%), and other (5%). This function appears unrelated to their description of the role they fulfilled in the MHPSS programme. Variations on terms such as “provision”, “planning” “facilitating” of psychosocial support services were used across the function categories. Terms such as “coordination” and “responsible” were primarily used by management staff, “psychosocial support” in combination with “medical”, “first aid” or “ambulance” mostly by volunteers.

### About the MHPSS programmes

Almost half of the MHPSS programmes (45%) were implemented in the wake of a natural disaster (i.e. flooding, earthquake, volcanic eruption). Approximately one quarter (28%) followed a terrorist act, a shooting, or a large-scale violent conflict. The other programmes were directed at populations confronted with accidents, including several plane crashes, fires, bus, and boat accidents. The number of deadly casualties in the events where the programme was carried out varied between none (12.5%), up to 25 (30%), between 25 and 100 (17.5%), between 100 and 1,000 (25%) and more than 100,000 (7.5%) (three respondents did not provide a number of casualties). Psychosocial services were provided to a general public of adults (95%) and children (82.5%). In most programmes (60%) the local community was the main target group of beneficiaries. A portion of the programmes provided services to refugees, migrants and internally displaced persons (15%).

In Table 1 the items per domain are listed, together with distributional information.

With respect to the planning and delivery system, fewer than half of the MHPSS programmes worked with a multi-agency planning group. However, in over two thirds of the programmes the programme coordinators reported good cooperation with other actors, mapping of existing psychosocial services, and involvement of trauma experts, local individuals, and politicians or government officials was achieved. In more than half of the programmes an overall emergency plan was available and the response was built upon existing guidelines. Psychosocial care plans were tested through exercises in fewer than half of the programmes.

According to the programme coordinators, over two thirds of the measures and interventions applied in the context of the MHPSS programmes involved professional treatment for acute stress or referral, information meetings with the affected, application of a stepped model of care, appropriate conditions or facilities for communal, cultural, spiritual and religious healing practices, distribution of psychoeducational leaflets, consideration of needs of particular vulnerable groups, and financial assistance. In more than half of the programmes site visits, memorial services, legal advice, and a telephone helpline were provided. Fewer than half of the programmes included an integrated co-ordination point for the long-term coordinated provision of aftercare or mental health complaints assessments.

Generally, observance of the essential psychosocial principles was scored positively by programme coordinators. Overall, they assigned higher scores to the importance of the essential principles compared to the actual level of success achieved in promoting a sense of safety, calmness, connectedness to others, self and community-efficacy, and hope.

In most cases the programme coordinators gave positive scores to the various general evaluation criteria. The highest programme scores were given for equal treatment of affected people and the appropriateness of measures and interventions. The degree to which the overall preparedness plan was helpful during the response, and the efficiency of the programme in reaching vulnerable groups both yielded somewhat lower scores.

### Step 1. Testing of constructs

S3 File contains the results of three analyses. The first analysis, carried out to confirm the presence of the four latent constructs, suggests that the preselected items actually load on three of the constructs. Six of the ten planning and delivery system items load on their latent construct (P < 0.05), with three other items slightly exceeding the threshold (P < 0.10). The remaining item (PD_6) seems to conflict with another emergency plan item (PD_7). Only three of the items assigned to measures and interventions applied load on this second construct (P < 0.05). This is also the case with seven essential psychosocial principles and eight general evaluation criteria items in relation to their respective constructs (P < 0.05).

These findings encouraged us to further explore a solution with three latent constructs. Since measures and interventions applied do not cluster together it is not logical to treat them as a construct. There are two items that could be part of the planning and delivery system (which pertain to coordinated cooperation between a variety of stakeholders) as well: integrated co-ordination point for long-term (MI_2); and co-ordination centre for aftercare (MI_14). Adding both items to the construct increases the internal consistency reliability of planning and delivery system from 0.79 to 0.82. In the second analysis, nine of the twelve items, including the newly-added coordination items, load on the adjusted construct (P < 0.05). No coefficient is produced for emergency plan item PD_6 and the PD_7 coefficient seems overestimated. The two other constructs perform better with less items. The seven essential psychosocial principles items (Cronbach’s alpha 0.75) and the eight general evaluation criteria items (Cronbach’s alpha 0.82) all load on their constructs (P < 0.05). In none of the three constructs does additional removal of items improve internal consistency reliability.

In order to further examine the problem with items PD_6 and PD_7 we conducted a third analysis but now without item PD_7. The eleven remaining items (including item PD_6) all load on the planning and delivery system construct (P < 0.05). The correlation between the 12-item construct average and the 11-item construct average is nearly perfect (*r* = 0.993; P < 0.001; N = 40). With this correlation and the higher internal consistency reliability in mind we prefer the 12-item over the 11-item construct. The three constructs are summarized in Table 2.

**Table 2:** Three constructs.

Item	Construct
	***Planning and delivery system (Cronbach’s Alpha 0*.*82; 12 items)***
PD_1	Multi-agency planning group
PD_2	Politicians or government officials involved in planning group
PD_3	Local individuals involved in planning
PD_4	Trauma experts involved in planning group
PD_5	Good cooperation with other actors
PD_6	Psychosocial care plan to use in emergencies
PD_7	Overall emergency plan
PD_8	Build upon existing guidelines
PD_9	Existing psychosocial services fully mapped
PD_10	Psychosocial care plan tested through exercises
MI_2	Integrated co-ordination point for long-term
MI_14	Co-ordination centre for aftercare
	***Essential psychosocial principles (Cronbach’s Alpha 0*.*75; 7 items)***
EP_1	Successful in providing safety
EP_2	Successful in promoting connectedness
EP_3	Successful in promoting a sense of calmness
EP_4	Successful in promoting self and community efficacy
EP_5	Successful in igniting hope
EP_9	Importance of promoting self and community efficacy
EP_10	Importance of igniting hope
	***General evaluation criteria (Cronbach’s Alpha 0*.*82; 8 items)***
GE_1	Responsive to needs and problems
GE_2	Overall preparedness plan helped to respond
GE_3	Effective in addressing needs and problems acute phase
GE_4	Effective in addressing needs and problems recovery phase
GE_5	Efficient (invested resources in relation to people assisted)
GE_6	Efficient in reaching vulnerable groups
GE_7	Appropriateness given circumstances
GE_8	Contribute to safety affected people

### Step 2. Modelling of associations

In step 2 we used the average item scores per construct (Table 2) to operationalize the domains planning and delivery system (12 items; mean 0.61, min-max 0.00–1.00, IQR 0.33), essential psychosocial principles (7 items; mean 3.94, min-max 2.00–5.00, IQR 0.64) and general evaluation criteria (8 items; mean 7.78, min-max 3.63–10.00, IQR 1.63) and to analyse the relations between these three domains, also in relation the twelve remaining measures and interventions. Since the measures and interventions do not load on one construct it is not suitable to calculate a mean score. Instead, we decided to make a distinction between the *number* and *nature* of measures and interventions applied within a programme.

We tested three models (Fig 1) using the mean domain scores and the number of applied measures and interventions (12 items; mean 6.65, min-max 1.00–12.00, IQR 3.50). The result of the SEM analysis is shown in Table 3. Relation a was significant in model A (P < 0.01); a higher score on planning and delivery system is accompanied by a larger number of measures and interventions applied. In model B the relations a (P < 0.01), d (P < 0.01) and e (P < 0.001) were significant, confirming the relevance of a more developed planning and delivery system for the number of measures and interventions applied, and for the domain scores of essential psychosocial principles and general evaluation criteria. These effects were sustained in model C after removal of the relations b and c. The goodness of fit statistics improved in each subsequent model tested; model C is the best model (Table 3). Modification indices did not suggest any paths to be added or removed to further enhance model fit.

**Table 3:** Structural equation modelling: testing three models.

	Model A			Model B			Model C		
	Coefficient	SE	P	Coefficient	SE	P	Coefficient	SE	P
MI_sum ← PD_mean (*relation a*)	4.713	1.420	.001	4.713	1.420	.001	4.713	1.420	.001
Constant	3.849	.971	.000	3.849	.971	.000	3.849	.971	.000
EP_mean ← MI_sum (*relation b*)	.032	.037	.378	-.022	.037	.546	-	-	-
EP_mean ← PD_mean (*relation d*)	-	-	-	1.150	.370	.002	1.044	.327	.001
Constant	3.703	.269	.000	3.357	.265	.000	3.271	.224	.000
GE_mean ← MI_sum (*relation c*)	.132	.072	.067	-.014	.065	.826	-	-	-
GE_mean ← PD_mean (*relation e*)	-	-	-	3.077	.647	.000	3.010	.570	.000
Constant	7.883	.531	.000	6.960	.463	.000	6.904	.390	.000
Chi-square (df, P)*	26.487 (3, .000)			.133 (1, .715)			.545 (3, .909)		
RMSEA (lower-upper) / PCLOSE	.454 (.305-.620) / .00			.000 (.000-.309) / .73			.000 (.000-.108) /.92		
CFI / TLI	.31 / .00			1.00 / 1.00			1.00 / 1.00		
AIC / BIC	400.933 / 415.672			378.579 / 396.593			374.991 / 389.729		

*Note*. EP_mean = Mean score essential psychosocial principles (7 items); GE_mean = Mean score general evaluation principles (8 items); MI_sum–Number of measures and interventions applied (12 items); PD_mean = Mean score planning and delivery system (12 items).

* LR test of model vs. saturated

To examine the relevance of the nature of distinct measures and interventions in relation to the three domain scores, we tested model A twelve times, each time with a different item from the items that remained after step 1 (S4 File). The chance that information meetings with the affected and site visits are organized, that needs of particular minorities or other vulnerable groups are considered, and that appropriate conditions/facilities are provided for communal, cultural, spiritual and religious healing practices is larger in programmes with more developed planning and delivery systems (*relation a*). Programmes with appropriate conditions/facilities for communal, cultural, spiritual and religious healing practices, focusing on needs of minorities and vulnerable groups, and incorporating a stepped care model, score higher on the essential psychosocial principles (*relation b*). Programme coordinators assigned a higher average general evaluation score when the programme includes appropriate conditions/facilities for communal, cultural, spiritual and religious healing practices, a stepped care model and psychoeducational leaflets (*relation c*). Mental health complaints assessments, telephone helplines, memorial services, professional treatment for acute stress or referral, financial assistance and legal advices are not associated with planning and delivery system, general evaluation criteria and essential psychosocial principles scores (*relations a*, *b and c*).

## Discussion

In this article we presented a methodology to operationalize the quality of MHPSS programmes based on a theoretical framework. By combining elements from different quality domains and by modelling the associations between the domains we could learn more about the structure, process and outcome of MHPSS programmes. Although the study is not devoid of limitations (expounded upon in the section following), our exploratory approach enabled us to measure the multi-faceted quality concept in the context of post-disaster MHPSS programmes. In our analysis of items, measured in a sample of 40 programmes, a priori four theoretical domains were operationalized. For three of the domains our data showed to empirically cluster in a coherent way. It appears possible to assess and compare the quality of MHPSS programmes in different settings and moments in time.

The study corroborates the gap between MHPSS norms and practices described by Te Brake and Dückers [28], particularly in relation to the essential psychosocial principles which belong–together with the general evaluation criteria–to the more subjective quality domains criteria of the four. Despite consensus on the importance of the essential principles in the context of the MHPSS programme, there is room for improvement when it comes to their practical implementation. Witteveen et al. observed the variation in adherence to evidence-informed guidelines at the level of European regions [19]. The variation in the developmental status of planning and delivery systems at the regional level was examined in greater detail in a study that confirmed the positive relation between system developmental status and a compilation of socio-economic country characteristics [20]. The analysis described in the present study also points at variation, this time however not at the level of individual professionals, countries, or regions but at the level of community programmes. The programmes generally score fairly high on the following: involvement of trauma experts, local individuals, and politicians or government officials in the planning group; professional treatment for acute stress or referral; information meetings with the affected; stepped care; and conditions or facilities for communal, cultural, spiritual and religious healing practices. The scores are lower for programme components such as a multi-agency planning group, coordination of (long-term) aftercare services, and the testing of psychosocial care plans. Apparently, there is room for improvement in collaboration, integration, and learning in the planning and delivery of MHPSS services.

As could be expected, in programmes encompassing richer planning and delivery systems, a larger number of measures and interventions from evidence-informed guidelines was applied. Programme coordinators in such programmes provide more positive self-evaluations, i.e. with respect to the general evaluation criteria, and the realization of essential psychosocial principles at the community level post-disaster. Some measures and interventions are more likely to be applied in programmes with more evolved planning and delivery systems, yet for a variety of measures and interventions the chance of being applied is not linked to planning and delivery system status, nor to coordinator perceptions concerning psychosocial principles and evaluation criteria.

The programme inquiry suggests that a MHPSS programme can serve as a transportation vehicle for the essential psychosocial principles. This is relevant given the criticism Benedek and Fullerton directed at the essential principles of Hobfoll and colleagues [35]. Hobfoll acknowledged that the possible working mechanisms and means of transportation of the principles received ample attention; an accompanying model with “passageways and obstacles” for the realization of the principles is however missing [26]. Ideally, a programme serves as a bridge between the temporary project organization in the wake of an event on the one hand, and longer-term regular healthcare capacity and other professional services on the other. MHPSS programmes can serve as a passageway and as a means for overcoming obstacles.

### Practical implications

Strengthening the structure of programmes–planning and delivery systems but also the capacity and skills of professionals and volunteers–is a route to increase the possibility of services to affected communities which encompass fitting measures and interventions. We recommend that support tools, education and training, aimed at standardization, go beyond the content of evidence-based diagnostic or therapeutic knowledge and interventions; specific guidance concerning the multi-organizational, inter-professional challenges awaiting responsible governments, planners, providers, and evaluators from a quality improvement perspective should also be given. Since disaster contexts will differ, and needs and problems of affected people develop over time, the organization and the composition of the programme should be able to adapt [36]. Instruments to support tailoring on behalf of the realization of MHPSS imperatives must be welcomed, especially when they combine MHPSS planning with evaluation [11,36,37]. Occasional evaluations of a programme are helpful to verify expectations, to ensure local needs and problems are actually addressed, and to promote learning during the implementation of the programme. We consider the instrument to assess the overall quality of MHPSS programmes presented in this article a major contribution to the standardization, monitoring, evaluation and overall improvement of programmes, potentially leading to a strengthening of quality assurance and effective resource management. The instrument and the items are formulated in such a way that they can be used in a variety of post-disaster situations, wherever MHPSS programmes are planned, delivered, or evaluated. Responsible stakeholders and decision-makers should welcome instruments like these as they can increase their opportunity to manage programmes based on structured empirical data rather than merely impressions.

### Further research

In our view, the development and testing of tools, educational curricula, and training schemes to accommodate the practical solutions mentioned above could benefit from additional research on a number of topics, contributing to a better understanding of MHPSS programme management. We recommend more qualitative and quantitative studies of the separate domains and their components, as well as interactions between them. Further, it is meaningful to learn more about the interrelation between contextual characteristics of the disaster setting and the programme. Which environmental features help or hinder implementation of a programme and particular components? Socio-economic country characteristics matter [20], but little is known about the question of how this works. And inversely: what is needed to tailor a programme to different country settings, with different healthcare systems and institutional characteristics, particularly when a crisis or public health risk extends beyond country borders. Evaluation of an integrated programme, locally implemented, covering MHPSS but also broader public health and safety topics can guide local quality management and may well suffice in a localized incident. However, more complex transboundary crises such as the refugee crisis, regional flooding or pandemics actually require cross-national coordination and programmes with multilateral planning and delivery; measures and interventions to address problems that extend beyond national borders, jurisdictions and conflicting interests also need to be developed.

Our analysis was theory-driven. The data we examined clustered in a way that fits the theoretical framework. There can be reasons for testing alternative frameworks and models, but nevertheless, it would be a most interesting exercise to link programme features to outcomes at the level of affected individuals or populations. The relation between a programme and health and well-being is complex and challenging to study. We are confronted with limited cases, limited material for comparison, many factors we cannot control for, and an abundance of possible interactions. Novel approaches and instruments are necessary to understand MHPSS programmes, and also to learn more about the role individual professionals and trained volunteers fulfil during a programme. Ultimately, the work is done by people, working within a programme’s interdisciplinary surroundings. Our study and the tool contribute to the knowledge base; we must however underline the relevance of research that goes further, and links knowledge about elements and working mechanisms of programmes, to practically equipping professionals and trained volunteers in an optimal fulfilment of their roles.

All in all, our understanding of MHPSS programmes would benefit from research on varying event types in different populations of beneficiaries, communities, countries, and world regions. Even studies in sectors outside MHPSS, where temporary multidisciplinary programmes are designed and implemented on behalf of groups of people, can be informative.

### Strengths and weaknesses

This study has several strengths. To our knowledge it is the first time multiple programmes have been evaluated in a similar manner against the background of a theoretical framework. The measurement instrument proved to be an effective way to gather information from different events in different situations in a systematic way, and allowed us to study patterns across programmes and programme domains.

A number of weaknesses need to be addressed. The measurements reflect the perspective of single respondents. This proved helpful in testing the clustering within datasets and relations, and can also be useful for improvement purposes, but it is still only one opinion from each programme. Approaching a programme coordinator to provide data is a logical source, but vulnerable to confirmation bias. Since scores on planning and delivery system and measures and interventions are concretely tied to actual structures or activities, these domains would seem to be relatively well protected. The other two domains are based on unanchored response scales and are therefore more vulnerable, though the instrument provides some protection by requiring respondents to do a careful substantive review before they rate these items. Whether from this kind of bias or from generally shared strong values, the importance scores appear higher and more skewed than the success scores and could possibly dilute the effect of items that more truly represent programme outcomes.

Given the likelihood that different stakeholders will view the quality of a programme from different perspectives it is preferable to also involve other stakeholders, particularly beneficiaries, in addition to gathering the indispensable input from programme coordinators. Including more viewpoints allows the opportunity to illuminate variation. Moreover, subjective views are ideally complemented by objective observations. Regarding the outcome of a programme, it is meaningful to look further than the perceived realization of psychosocial principles, and to collect information on changes in well-being, risk and protective factors, and psychopathology within the population–bearing in mind that we must remain critical about the extent to which these “outcomes” can really be attributed to the programme and not to other developments or circumstances. A further shortcoming is that our analysis does not take into account changes over time. This is a limitation as aftercare will, or even should, be anticipated as needs and problems evolve. The quality of a programme can score high at one moment and lower at another. Particularly in the first period following a large disaster the population is fluid; later specific issues of particular target groups are typically addressed in the communities where the affected reside. The survey tool referred to in this study can be used for this purpose. Finally, the sample size is far from optimal. It did not allow for more advanced analysis, nor did it accommodate expanding the number of items (e.g. the management of volunteers, advanced therapeutic interventions) or even domains.

### Conclusions

In this study we describe an approach to coherently measure the quality of post-disaster MHPSS programmes, focusing on different domains and items derived from theory, guidelines, and a wealth of practical experience. The interrelations between the domains confirm the assumption that more evolved planning and delivery systems are accompanied by a higher adoption of evidence-informed measures and interventions, and score higher on a variety of general evaluation criteria and on the importance and realization of essential MHPSS principles. The findings suggest that high-quality programmes serve as “transport vehicles” for the realization of these principles at the community level. Moreover, community programmes can serve as a passageway towards professional care and support. Temporary organizations, set up to accommodate new disaster-driven needs and problems, form ideally in the end a bridge to regular services. Further research is necessary to validate and expand the findings herein and to learn more about success factors and obstacles for the implementation of programmes in communities confronted with extraordinary adversity.

## Supporting information

S1 File PsyQual—MHPSS programme evaluation tool. (PDF)

S2 File Dataset. (ZIP)

S3 File Generalized structural equation modelling. Step 1. Testing of constructs.
(DOCX)

S4 File Generalized structural equation modelling. Step 2. Modelling of associations.
(DOCX)
