# Supplementary material for: Measuring and modelling the quality of 40 post-disaster mental health and psychosocial support programmes
Source: PLoS One. 2018 Feb 28;13(2):e0193285. doi: 10.1371/journal.pone.0193285 (PMC5830995; doi:10.1371/journal.pone.0193285)
Supplement: S1 File — (PDF) [file pone.0193285.s001.pdf]

PSYQUAL

Measuring quality of psychosocial programming

-Full version for larger events-

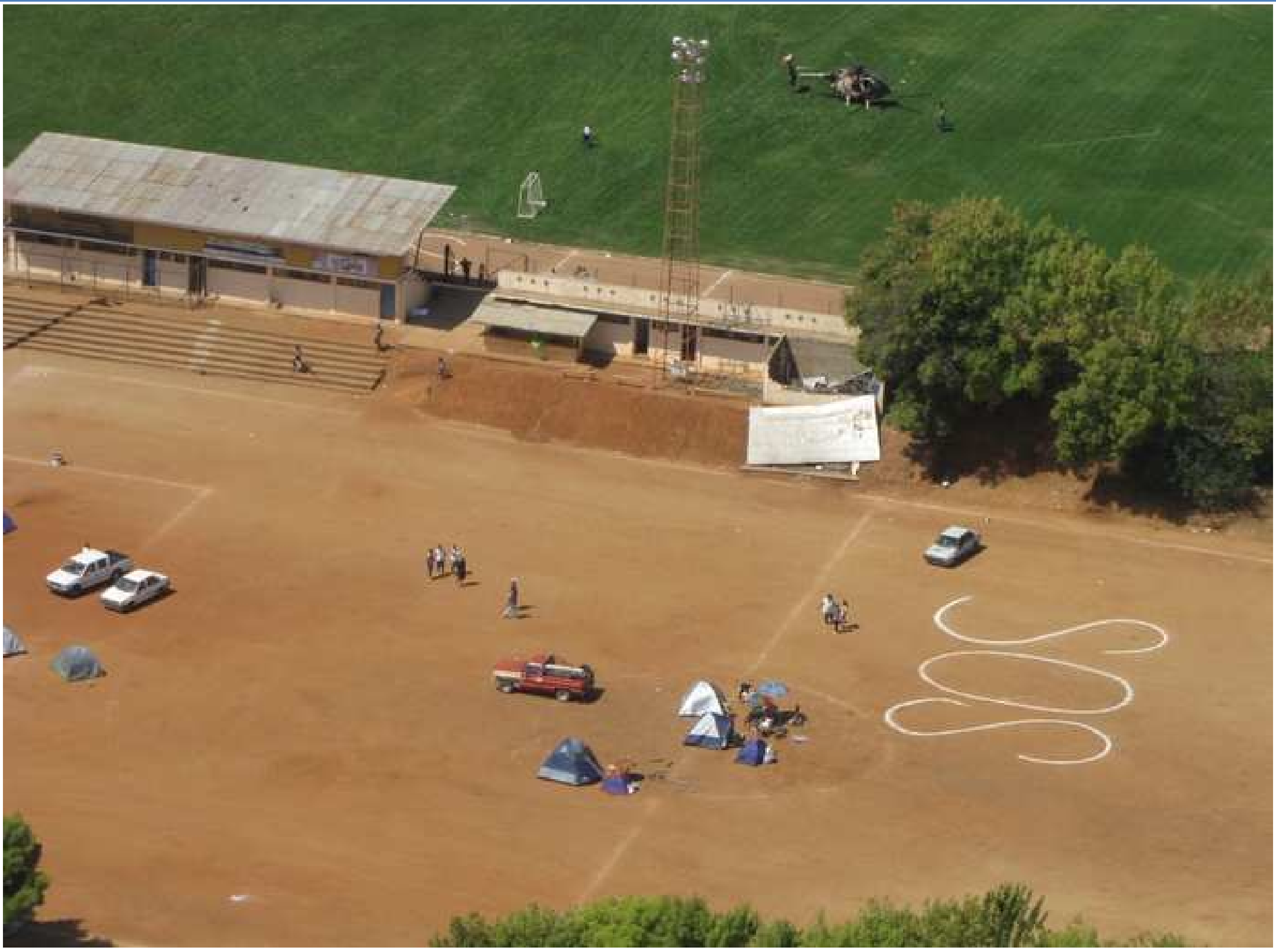

Academic Medical Center – Amsterdam

Deliverable 3 - WP 3

Authors:
Sigridur Bjork Thormar
Miranda Olff

PART 1. Programme content

Date of filling in the questionnaire: ____/____/_____(DDMMYYYY)

1. Name of your organization:____

2. What is the role of the organization in disaster response:____
____

3. Your function:
- Management (staff)
- Desk officer (staff)
- Program manager (staff)
- Volunteer (name your specific function):____
- Other:______________________________

The first section of this tool refers to the ***event and your interventions***.
Kindly fill in the information as detailed as possible by either picking the appropriate choice or by filling in the qualitative sections.
If something does not fit your event and/or intervention please write clarifications to the side of the document

4.	Year of the event	(YYYY)	
5.	Location of the event:	Country	City

6. What was the nature of the event?
   a. Natural disaster
   b. Event with intention to cause harm
   c. Human error
   d. Accident
   e. Financial crisis
   f. Other

7. Please describe shortly the nature, year and impact of the event. (Examples: Flooding 2005 (15 villages affected and 3 regions affected), Tsunami 2004 (hundreds of xx citizens in xx, Thailand and Sri Lanka affected), Avalanche 2009 (one village of 300 people affected).

____
____
____
____
____
____
____
____

8. What was the **approximate** number of:

a. Casualties (deaths) ____________
b. Severely injured survivors ____________
c. Somewhat injured survivors ____________
d. Non-injured survivors ____________
e. Families and friends seeking psychosocial support ____________
f. Missing persons in the beginning ____________

9. What was the **approximate** amount of other losses involved? %

a. Property ____________
b. Livelihood ____________
c. Livestock ____________

10. Who provided Psychosocial Support during this event?

a. One organization: Please specify. ____________
b. Many organizations: Please list them all. ____________

11. Was it clear who was professionally leading the intervention and had responsibility for it?

Yes	No

If no, why was it not clear?

12. What were the target groups of beneficiaries of the PSS ? (please mark all relevant groups)

a. Local community
b. Foreigners (Example: bus accident with tourists)
c. Both (e.g. train accident)
d. Citizens of your own country involved in an event abroad

13. Within these groups, for whom did you provide PSS?

a. General public
    1. Adults
    2. Children
    3. Elderly
    4. Other groups e.g. refugees, please specify:

b. Emergency responders staff/volunteers
    1. Fire fighters
    2. Ambulance services
    3. Crisis managers
    4. Other professional rescue teams
    5. Emergency responders staff/volunteers

c. Others
    1. Military forces
    2. Police
    3. Others

____

____

____

14.	What kind of PSS interventions were provided for the affected population? Please mark all interventions that were carried out.	Location of intervention	Duration (start/end)
a.	Reception centre for survivors		
b.	Reception centre for families and friends		
c.	Rest centre		
d.	Family reunions		
e.	PSS in hospitals (especially for this event)		
f.	PSS at morgue and during death notifications		
g.	PSS provided by mobile teams (e.g. at people's homes in relation to flooding or PSS integrated into search teams or medical support teams)		
h.	Distribution of psychoeducation leaflets		
i.	Information points in shelters/receptions centers/camps etc.		
j.	Information meetings with the affected communities		
k.	Evacuation centre		
l.	PSS at school or kindergarten		
m.	Play and recreational activities for children and adolescence		
n.	PSS trainings and/or information for teachers		
o.	PSS trainings and/or information for general practitioners		
p.	Telephone helpline		
q.	Website for the affected people		
r.	PSS integrated into shelters/reception centers/camps etc.		
s.	PSS integrated into evacuation center		
t.	Memorial services		
u.	Site visits		
v.	Co-ordination centre for aftercare:		

w. One stop shop (mostly doing referrals to different forms of counseling)

x. Long term humanitarian assistance center

y. A stepped model of care was used (psychological first aid, psychosocial support and clinical support was given to those in need)

z. Other:

15.	Within these interventions **for beneficiaries** how important were the following elements (0=not important 5=very important) and to which degree do you think you succeeded in reaching the aim (0=not successful 5=very successful)	Importance (0-5)	Successf (0-5)
a.	**Providing safety** : e.g. safe places, information on event and missing persons, protection		
b.	**Connectedness:** e.g. activating social support networks, family reunions		
c.	**Calmness:** e.g. psychoeducation and protection from too much stress		
d.	**Self and community efficacy:** e.g. information, coaching, helping to make decisions and take action, involvement into planning of future interventions		
e.	**Igniting hope:** e.g. coaching for future steps, providing further help and aftercare		

16. What sort of support did you provide for staff and/or volunteers

	For staff	For volunteers
Debriefings (please describe it shortly)		
a. One-on-one support		
b. Demobilizations		
c. On scene support		
d. Co-ordination point for aftercare		
e. Certificates and/or other forms of positive feedback		

f. Other interventions, please specify:

17. Within these interventions for **staff/volunteer** how important were the following elements (0=not important 5=very important) and to which degree do you think you succeeded in reaching the aim (0=not successful 5=very successful)

	Importance (0-5)	Successful (0-5)
**Providing safety:** e.g. safe places, information on event and missing persons, protection		
**Connectedness:** e.g. activating social support networks, family reunions		
**Calmness: e**.g. psychoeducation and protection from too much stress		
**Self and community efficacy:** e.g. information, coaching, helping to make decisions and take action, involvement into planning of future interventions		
**Igniting hope:** e.g. coaching for future steps, providing further help and aftercare		

The second section of this tool refers to the **PREPAREDNESS PHASE**. Kindly fill in the information as detailed as possible.

18. Did you have a psychosocial care plan to use in emergencies? Yes No

19. Was it a part of an overall emergency plan? Yes No

20. Did you build your psychosocial care plan from existing guidelines on the provision of psychosocial care in emergencies?

a. Yes, completely
Name of guidelines:

____

____

b. Yes, partially
Name of guidelines:

____

____

c. No
d. Other, namely:

21. Before the event had the psychosocial care plan been tested through exercises?

a. Yes, regularly
b. Yes, but last time was in: Year (take out BUT)
c. No, it had not been tested before
d. I don´t know if they were tested earlier
e. Other, namely:

22. Was there a multi agency care planning group set up before hand?

a. Yes
b. No
c. Other, namely:

23. Did this group include mental health professionals with expertise in traumatic stress?

a. Yes
b. No
c. Other, namely:

24. Was there good co-operation with other key agencies that may not have been a part of the planning group?

a. Yes
b. No
c. If no, please explain why not:

25. Were local individuals who were aware of local cultures and particular communities involved in the psychosocial care planning group?

a. Yes, they were well represented
Please give examples:

b. Yes, but it could have been better

c. No they were not
If no, please explain why not:

26. Were politicians/government officials involved in the planning group?

a. Yes
b. No

27. Were existing psychosocial services fully mapped in your area and incorporated into the psychosocial care plan (or guidelines)?

a. Yes they were fully mapped and incorporated into the psychosocial guidelines
b. Yes, they were fully mapped but not yet incorporated into the psychosocial guidelines
c. No, they had not been mapped

28. Had there been a pre-recruitment of care providers (staff and volunteers) to allow for screening of suitability before being accepted?

a. Yes, it was all done as a part of preparedness

b. No, it was all done in the first 24 hours

c. No, it was done in the first 24 hours but then continuously when a new staff or volunteer members joined

d. Not in the emergency phase, but later when hiring staff

e. No, there were no measures taken to screen for suitability

29. If there was screening, what were your screening criteria?

____

____

____

____

30. Did you work with volunteers for your intervention?

a. Yes we did

b. No we did not

31. If so were they:

a. predefined

b. recruited during the intervention

c. both predefined and recruited

32. What kind of volunteers did you have?

a. Volunteers associated with my organizations and are unpaid

b. (Spontaneous) volunteers that came because of an advertisement for assistance due to the crisis

c. Both organizational volunteers and spontaneous volunteers. Please try to indicate the percentage of spontaneous volunteers in the response. __________ %

33. If you did work with volunteers how easily available were they?

Very unavailable 0 1 2 3 4 5 6 Very available

34. Had you pre-trained your staff or volunteers in provision of psychosocial support prior to the disaster? **<u>(please put an X for either staff or volunteer or both if you worked with both)</u>**

	For staff	For volunteers
a. Yes, we had a very good training programme in place that was being carried out regularly		
b. Yes, we had a very good training programme in place but we had not yet started using it		
c. Yes, we had a training programme but not a very good one but we used it		
d. Yes, we had a training programme but not a very good one and we did not use it		
e. No, training took place before the event		

35. If training took place, please describe the elements and **approximate amount** of the training;

36. Material used for the training (full reference if possible);

37. Who provided the training (type of professional or organization)

38. If your training has changed in amount or content over time please elaborate in what way?

39. Do you feel that the content and level of the training programme was tailored to correspond with the roles and responsibilities of the providers of psychosocial care?

a. Yes, very much so
b. Yes, to some extent
c. Not completely
d. No, not at all

40. Can you give example of success with training?

The third section of this tool refers to the **RESPONSE PHASE**. Kindly fill in the information as detailed as possible.

41. How quickly after the event were you able to start your intervention?

a. The day the event occurred
b. Within 1-4 days
c. Within 5-7 days
d. Other ____________

42. How well do you feel your overall preparedness plan worked to respond in the actual crisis?

Not well at all 0 1 2 3 4 5 6 Very well

43. How did you identify the PSS needs after the event?

a. There was a PSS assessment done
Please explain who did the assessment:
b. The PSS crisis management gave instructions about who to serve
c. Other

44. Was your psychosocial approach multidisciplinary?

a. Yes. Can you describe in what way?

b. No. Why not?

45. Had there been a pre-recruitment of care providers (staff and volunteers) to allow for screening of suitability before being accepted?

a. Yes, it was all done as a part of preparedness
b. Yes, it was all done in the first 24 hours
c. Yes, it was done in the first 24 hours but then continuously when a new staff or volunteer member joined
d. No, there was no time to screen or select, we took whomever we could use.

46. Were there efforts put into providing accurate information regarding the situation to address the concerns of individuals affected?

Yes, we:

a. Could give accurate information about the event, the missing persons and the future steps by establishing communication link between the affected and authorities, experts and other relevant information providers

b. Held information meetings together with authorities and experts as means to help people understand the event as well as the necessary steps to be taken and the further support that was to be provided

c. Held information meetings as means to normalize the psychological reactions for the affected.

d. We provided educational leaflets with information about responses to traumatic events, helpful coping strategies and where to seek further assistance.

e. We launched a website with information about psychosocial issues.

f. We contributed to an existing website launched by another party (please mention who):

g. Other measures taken to provide information were: the following (<-suggest: delete)(please write them out):

h. No we had no means to do that
Please explain why not:

____
____
____
____
____

47. Do you feel that information was flowing properly – so that details of telephone lines, websites etc. reached all who needed them?

a. Yes, the flow of information was very good
b. Yes, the flow was relatively good
c. Not good enough
d. Not good at all

48. Comments to question 47?

____
____
____
____

49. Was there a telephone helpline set up staffed by trained personnel that provided emotional support to those directly or indirectly affected by the event?

a. No, we had no means to do that
b. Yes, it was set up immediately within the first 24 hours
c. Yes, but later than 24 hours. Please specify when:

d. Yes, but not within our intervention (Please name who set it up):
e. Other ____________

50. Was there an element of restoring family links set up?

a. Yes it was set up within 24 hours
b. Yes it was set up after________ days
c. No there was no need for such services

51. Comments to question 50?:

____
____
____

52. Was there good cooperation between the family links setup and the police or DVI teams?

a. Yes very good co-operation
b. Yes but it could have been better. Please explain how:

____
____

c. No, there was not

The fourth section of this tool refers to the **<u>RECOVERY PHASE</u>**. Kindly fill in the information as detailed as possible.

53. How long did you continue your intervention?

54. Was the funding resource:

a. Sustainable till the end of the intervention
b. Temporary requiring us to gather new funding after ________ months.
c. Based on efficiency and could be revoked if the intervention was not meeting set aims.
d. Other

55. Was there ongoing governmental/authority provision of adequate funding to maintain a good psychosocial intervention that could be effectively delivered during the disaster?

a. Yes, there was good financial support from government or other type of authority
b. Yes, there was some support but more funding needed to be gathered
c. No, there was no follow up support in terms of finances

56. If yes, who funded the psychosocial care (e.g. local council/government/Red Cross or other NGO/EU/fund raising activities etc:

57. Was there any financial assistance put into place for the affected?

a. Yes there was
Please explain:

b. No there was none (suggestion: add: if not can you explain why not?)

58. Were there any services for legal advice put into place for the affected?
a. Yes there were
b. No there were no such services provided
c. No, but victims created their own legal advice group

59. How active do you feel your intervention was towards beneficiaries with high levels of distress:

Very reactive 0 1 2 3 4 5 6 Very proactive

60. Please explain:

___

___

___

___

61. If you feel your intervention was more pro-active, for how long did you remain proactive? (years/months/weeks/days)

____

62. Was there any professional treatment/services provided for those with acute stress disorder, severe acute post-traumatic stress disorder or other types of (pre-existing) mental health problems?

a. No, we provided no treatment

b. No, we provided no treatment but referred them to: predefined organizations in each part of the country. Please provide name and nature of organization:__________

c. Yes we provided treatment. Please describe:

i. What type of treatment was performed?

ii. Who performed this treatment?

d. Other

63. Did these services also apply to first responders?

a. Yes

b. No. Please explain why not:

____
____
____
____
____

64. Were general practitioners/local doctors aware of possible mental and physical symptoms that could be expressed by individuals after a traumatic event?

a. Yes very well. Please explain how this was done:

b. No not well enough. Please explain why not:

65. Was there any assessment/evaluation done with regards to levels of mental health complaints?

Yes	No

66. If yes, can you please describe that assessment? Evaluation procedure

___

___

___

67. Can you please summarize the findings of the assessment?

___

___

___

___

___

68. For which purpose was the assessment done?

___

___

69. Who did the assessment? (function of person)

___

___

70. What was assessed?

___

___

71. How often was it assessed?

___

___

___

72. Were memorial services/ceremonies or site visits planned in conjunction with those affected?

a. Yes, both memorial services and site visits were planned
b. Yes, memorial services were planned
c. Yes, site visits were planned
d. No, no such planning was made

73. Any other interventions planned together with the affected? Please specify

a. Yes they were. Please explain what was done:

b. They were planned but not in conjunction with those affected
Please explain why not:

c. They were not planned at all. Please explain why not:

____
____
____

74. Did the staff and/or volunteers receive ongoing **training**?

a. Yes, please specify for how long:

____

b. No, why not?

____
____

75. Did the staff and/ or volunteers receive ongoing **supervision and support** during the intervention?

Yes, please specify for how long (days/weeks/months)

____
____
____
____
____
____

76. If yes to previous question (otherwise skip) please describe who provided the supervision:

a. a local mental health professional (psychiatrist, psychologist, psychiatric nurse)

b. a local health professional (doctor/nurse/social worker)

c. a local counselor or trained peer supporter

d. other:

DELETE LAST OPTION IN TOOL FOR THIS QUESTION Q75 IN TOOL
Q76 in the TOOL has been deleted here

77. Was there any monitoring of possible secondary traumatization and burn out symptoms among staff and/or volunteers?

a. Yes. Please explain who did the monitoring and how it was done:

____
____
____
____

b. No there was no such monitoring

78. Did the intervention take into account the needs of minority or particularly vulnerable groups?

a. If yes, please provide examples:

____

____

b. Yes, but some better than others. Please provide examples:

____

____

____

____

c. No, not well enough

d. No, not at all

79. Was there care taken to provide appropriate conditions/facilities for communal, cultural, spiritual and religious healing practices?

a. Yes, this was done for every group we identified

b. Yes, for most but not for all

If so, why was that:

____

____

c. No, we did not take care of this aspect of the response

If no, please explain why not:

____

____

80. Was there a co-ordination point for long term care integrated?

a. Yes there was. Please describe it:

____

____

____

____

____

b. No it was not integrated

c. Other

PART 2. Programme quality

The questions in this part of the tool are to be answered after completion of part 1. Otherwise it is not possible to assign meaningful scores to quality criteria.

Need centeredness

81. To what extent was your psychosocial programme responsive to the needs and problems of affected individuals?
Not need-centred at all 0 1 2 3 4 5 6 7 8 9 10 Very need-centred

82. Please explain what should have been different:

Effectiveness

83. How well do you feel your overall preparedness plan worked to respond to the psychosocial consequences of the crisis?
Not well at all 0 1 2 3 4 5 6 7 8 9 10 Very well

84. Please explain what should have been different?

85. How effective was your programme in addressing the needs and problems of the affected individuals in the acute phase?
Very ineffective 0 1 2 3 4 5 6 7 8 9 10 Very effective

86. Please explain what should have been different:

87. How effective was your programme in addressing the needs and problems of the affected individuals in the recovery phase?
Very ineffective 0 1 2 3 4 5 6 7 8 9 10 Very effective

88. Please explain what should have been different:

89. Did the programme, in your opinion, promote self-efficacy/empowerment in **individuals**?

a. Yes, please explain to what extent and why

b. No, please explain

90. Did the programme, in your opinion, promote **community** efficacy/empowerment?

a. Yes, please explain to what extent and why

b. No, please explain

Efficiency

91. How efficient – invested resources in relation to people assisted – was your psychosocial programme?
Very inefficient 0 1 2 3 4 5 6 7 8 9 10 Very efficient

92. Please explain what should have been different:

93. Do you feel that your PSS program was able to reach vulnerable groups efficiently?

Very inefficient 0 1 2 3 4 5 6 7 8 9 10 Very efficient

94. Please explain what should have been different:

Timeliness/appropriateness

95. Do you feel the intervention started early enough?
Yes I feel it started early enough
No, I feel it should have started somewhat earlier
No I feel it should have started much earlier

96. If you feel it should have started earlier or much earlier, please explain why:

97. To what extent was the content of your psychosocial programme appropriate given the circumstances of the event?
Highly inappropriate 0 1 2 3 4 5 6 7 8 9 10 Very appropriate

98. Please explain what should have been different:

Safety

99. To what extent did the programme contribute to the safety of affected people?
Not safe at all 0 1 2 3 4 5 6 7 8 9 10 Very safe

100. Please explain what should have been different:

101. To what extent did the programme contribute to the safety of service providers/staff?
Not safe at all 0 1 2 3 4 5 6 7 8 9 10 Very safe

102. Please explain what should have been different:

Equity

103. To what extent were affected people treated equally by the programme (no differences regardless of gender, age, ethnicity, social-economic status)?
No equality at all 0 1 2 3 4 5 6 7 8 9 10 Very equal

104. Please explain what should have been different:

'Good practices'

105. Which interventions or programme elements do you consider indispensable for future events? (you can mention more than one)

'Bad practices'

106. Which interventions or programme elements should be left out in the future? (you an mention more than one)

Any additional feedback on content or structure of the questionnaire is most welcome
