# Supplementary material for: Measuring and modelling the quality of 40 post-disaster mental health and psychosocial support programmes
Source: PLoS One. 2018 Feb 28;13(2):e0193285. doi: 10.1371/journal.pone.0193285 (PMC5830995; doi:10.1371/journal.pone.0193285)
Supplement: S3 File — Step 1. Testing of constructs. (DOCX) [file pone.0193285.s003.docx]

**S3 File.** Generalized Structural Equation Modelling. Step 1. Testing of constructs.

In step 1 we used the data to operationalize the quality domains of a mental health psychosocial support programme, tested constructs and assessed their internal consistency reliability. Three analyses were conducted: (1) Hypothetical model (44 items, 4 constructs); (2) Based on analysis 1 and internal consistency reliability (27 items, 3 constructs); (3) Based on analysis 2 without item PD_7 (26 items, 3 constructs).

|  |  | **Analysis 1.** | | | | **Analysis 2.** | | | | **Analysis 3.** | | | |
| --- | --- | --- | --- | --- | --- | --- | --- | --- | --- | --- | --- | --- | --- |
| **Item** | **Description** | **Con-struct** | **Coef-ficient** | **SE** | **P** | **Con-struct** | **Coef-ficient** | **SE** | **P** | **Con-struct** | **Coef-ficient** | **SE** | **P** |
| PD_1 | Multi-agency planning group | PD10 | .856 | .403 | .034 | PD12 | .755 | .354 | .033 | PD11 | .869 | .411 | .034 |
| PD_2 | Politicians or government officials involved in planning group | PD10 | .927 | .448 | .038 | PD12 | 1.091 | .478 | .022 | PD11 | 1.039 | .484 | .032 |
| PD_3 | Local individuals involved in planning | PD10 | 1.275 | .619 | .039 | PD12 | .958 | .430 | .026 | PD11 | 1.480 | .753 | .049 |
| PD_4 | Trauma experts involved in planning group | PD10 | .828 | .431 | .054 | PD12 | .738 | .374 | .048 | PD11 | .853 | .428 | .046 |
| PD_5 | Good cooperation with other actors | PD10 | .792 | .400 | .048 | PD12 | .669 | .335 | .046 | PD11 | .825 | .402 | .040 |
| PD_6 | Psychosocial care plan to use in emergencies | PD10 | 2.387 | 1.676 | .154 | PD12 | NA | NA | NA | PD11 | 1.243 | .563 | .027 |
| PD_7 | Overall emergency plan | PD10 | 1.703 | .930 | .067 | PD12 | 4.455 | 3.743 | .234 | - | - | - | - |
| PD_8 | Build upon existing guidelines | PD10 | .971 | .432 | .025 | PD12 | .809 | .349 | .021 | PD11 | 1.221 | .571 | .032 |
| PD_9 | Existing psychosocial services fully mapped | PD10 | .717 | .381 | .060 | PD12 | .630 | .330 | .056 | PD11 | .739 | .366 | .043 |
| PD_10 | Psychosocial care plan tested through exercises | PD10 | 1.016 | .439 | .021 | PD12 | 1.043 | .423 | .014 | PD11 | 1.147 | .520 | .027 |
| MI_1 | Mental health complaints assessment | MI14 | .131 | .250 | .601 | - | - | - | - | - | - | - | - |
| MI_2 | Integrated co-ordination point for long-term | MI14 | 1.017 | .465 | .029 | PD12 | .911 | .373 | .015 | PD11 | .966 | .431 | .025 |
| MI_3 | Appropriate conditions/facilities for communal, cultural, spiritual and religious healing practices | MI14 | NA | NA | NA | - | - | - | - | - | - | - | - |
| MI_4 | Needs of minority or particular vulnerable groups taken into account | MI14 | 1.253 | .568 | .028 | - | - | - | - | - | - | - | - |
| MI_5 | Site visits | MI14 | 1.368 | .655 | .037 | - | - | - | - | - | - | - | - |
| MI_6 | Legal advice | MI14 | .204 | .252 | .419 | - | - | - | - | - | - | - | - |
| MI_7 | Financial assistance | MI14 | .477 | .288 | .098 | - | - | - | - | - | - | - | - |
| MI_8 | Stepped model of care | MI14 | .436 | .355 | .219 | - | - | - | - | - | - | - | - |
| MI_9 | Professional treatment for acute stress or referral | MI14 | .341 | .285 | .232 | - | - | - | - | - | - | - | - |
| MI_10 | Memorial services | MI14 | .207 | .300 | .490 | - | - | - | - | - | - | - | - |
| MI_11 | Information meeting with the affected | MI14 | .790 | .455 | .082 | - | - | - | - | - | - | - | - |
| MI_12 | Telephone helpline | MI14 | .229 | .296 | .439 | - | - | - | - | - | - | - | - |
| MI_13 | Psychoeducational leaflets | MI14 | .507 | .339 | .134 | - | - | - | - | - | - | - | - |
| MI_14 | Co-ordination centre for aftercare | MI14 | 5.699 | 10.548 | .589 | PD12 | 1.104 | .490 | .024 | PD11 | .926 | .452 | .040 |
| EP_1 | Successful in providing safety | EP10 | .585 | .198 | .003 | EP7 | .575 | .201 | .004 | EP7 | .584 | .196 | .003 |
| EP_2 | Successful in promoting connectedness | EP10 | .454 | .173 | .009 | EP7 | .461 | .172 | .008 | EP7 | .448 | .170 | .008 |
| EP_3 | Successful in promoting a sense of calmness | EP10 | .363 | .207 | .079 | EP7 | .436 | .208 | .036 | EP7 | .414 | .218 | .058 |
| EP_4 | Successful in promoting self and community efficacy | EP10 | .457 | .165 | .006 | EP7 | .479 | .166 | .004 | EP7 | .468 | .167 | .005 |
| EP_5 | Successful in igniting hope | EP10 | .715 | .219 | .001 | EP7 | .803 | .224 | .000 | EP7 | .768 | .221 | .001 |
| EP_6 | Importance of providing safety | EP10 | .323 | .201 | .108 | - | - | - | - | - | - | - | - |
| EP_7 | Importance of promoting connectedness | EP10 | .181 | .113 | .108 | - | - | - | - | - | - | - | - |
| EP_8 | Importance of promoting a sense of calmness | EP10 | .166 | .083 | .046 | - | - | - | - | - | - | - | - |
| EP_9 | Importance of promoting self and community efficacy | EP10 | .407 | .153 | .008 | EP7 | .350 | .156 | .025 | EP7 | .359 | .152 | .018 |
| EP_10 | Importance of igniting hope | EP10 | .618 | .122 | .000 | EP7 | .600 | .130 | .000 | EP7 | .620 | .132 | .000 |
| GE_1 | Responsive to needs and problems | GE10 | .807 | .154 | .000 | GE8 | .792 | .145 | .000 | GE8 | .797 | .142 | .000 |
| GE_2 | Overall preparedness plan helped to respond | GE10 | 2.222 | .381 | .000 | GE8 | 2.276 | .343 | .000 | GE8 | 2.213 | .342 | .000 |
| GE_3 | Effective in addressing needs and problems acute phase | GE10 | 1.769 | .384 | .000 | GE8 | 1.807 | .366 | .000 | GE8 | 1.800 | .362 | .000 |
| GE_4 | Effective in addressing needs and problems recovery phase | GE10 | .911 | .373 | .015 | GE8 | .947 | .371 | .011 | GE8 | .949 | .367 | .010 |
| GE_5 | Efficient (invested resources in relation to people assisted) | GE10 | .819 | .257 | .001 | GE8 | .788 | .250 | .002 | GE8 | .767 | .249 | .002 |
| GE_6 | Efficient in reaching vulnerable groups | GE10 | 1.001 | .352 | .005 | GE8 | 1.012 | .349 | .004 | GE8 | 1.043 | .344 | .002 |
| GE_7 | Appropriateness given circumstances | GE10 | 1.111 | .257 | .000 | GE8 | 1.124 | .246 | .000 | GE8 | 1.101 | .244 | .000 |
| GE_8 | Contribute to safety affected people | GE10 | .661 | .299 | .027 | GE8 | .610 | .294 | .038 | GE8 | .616 | .290 | .034 |
| GE_9 | Contribute to safety services providers/staff | GE10 | .572 | .317 | .071 | - | - | - | - | - | - | - | - |
| GE_10 | Affected people treated equally | GE10 | .329 | .324 | .310 | - | - | - | - | - | - | - | - |
|  |  |  |  |  |  |  |  |  |  |  |  |  |  |
|  | Covariance PD ↔ MI | - | .826 | .143 | .000 | - | - | - | - | - | - | - | - |
|  | Covariance MI ↔ EP | - | .413 | .216 | .056 | - | - | - | - | - | - | - | - |
|  | Covariance MI ↔ GE | - | .509 | .171 | .003 | - | - | - | - | - | - | - | - |
|  | Covariance PD ↔ EP | - | .377 | .209 | .072 | - | .388 | .189 | .040 | - | .323 | .201 | .108 |
|  | Covariance PD ↔ GE | - | .786 | .108 | .000 | - | .753 | .104 | .000 | - | .734 | .115 | .000 |
|  | Covariance EP ↔ GE | - | .454 | .172 | .008 | - | .380 | .180 | .035 | - | .406 | .176 | .021 |
|  |  |  | | | |  | | | |  | | | |
|  | **Information criteria** | AIC: 3322.715 / BIC: 3513.558 | | | | AIC: 2300.596 / BIC: 2420.507 | | | | AIC: 2265.962 / BIC: 2384.183 | | | |

*Note.* PD = Planning and delivery system, MI = Measures and interventions applied, EP = Essential psychosocial principles, GE = General evaluation criteria, AIC = Akaike's information criterion, BIC = Bayesian information criterion. PD and MI items are binary variables, EP and GE items are continuous variables. Analyses 1 and 2 did not converge after 20 iterations.
