# Supplementary material for: Measuring and modelling the quality of 40 post-disaster mental health and psychosocial support programmes
Source: PLoS One. 2018 Feb 28;13(2):e0193285. doi: 10.1371/journal.pone.0193285 (PMC5830995; doi:10.1371/journal.pone.0193285)
Supplement: S4 File — Step 2. Modelling of associations. (DOCX) [file pone.0193285.s004.docx]

**S4 File.** Generalized Structural Equation Modelling. Step 2. Modelling of associations

In step 2 we tested several models using calculated mean scores for the domains planning and delivery system (12 items; PD_mean), essential psychosocial principles (7 items; EP_mean) and general evaluation criteria (8 items; GE_mean) (based on the results of step 1; see Supplement File 3). We analysed the relations between the three domains, also in relation to the *number* and *nature* of the twelve remaining measures and interventions applied. A table with the results of the analyses focusing on the number of measures and interventions is presented in the main text. To examine the relevance of the nature of distinct measures and interventions, model A was tested twelve times. Model A consists of three relations: positive effect of planning and delivery system on chance a measure or intervention is applied (*relation a*); positive effect of an applied measure or intervention on essential psychosocial principles (*relation b*); positive effect of an applied measure or intervention on general evaluation criteria (*relation c*). Each of the twelve analyses was conducted with a different measure or intervention: MI_1 = Mental health complaints assessment; MI_3: Appropriate conditions/facilities for communal, cultural, spiritual and religious healing practices; MI_4 = Needs of minority or particular vulnerable groups taken into account; MI_5  = Site visits; MI_6 = Legal advice; MI_7  = Financial assistance; MI_8 = Stepped model of care; MI_9 = Professional treatment for acute stress or referral; MI_10 = Memorial services; MI_11 = Information meeting with the affected; MI_12 = Telephone helpline; MI_13 = Psychoeducational leaflets.

| # | *Relation a* | Coef-ficient | SE | P | *Relation b* | Coef-ficient | SE | P | *Relation c* | Coef-ficient | SE | P | AIC / BIC |
| --- | --- | --- | --- | --- | --- | --- | --- | --- | --- | --- | --- | --- | --- |
| 1 | MI_1 🡨 PD_mean | -.433 | .760 | .955 | EP_mean 🡨 MI_1 | .120 | .219 | .584 | GE_mean 🡨 MI_1 | .118 | .470 | .802 | 250.749 / |
|  | Constant | -.330 | .497 | .507 | Constant | 3.867 | .131 | .000 | Constant | 8.702 | .275 | .000 | 263.417 |
| 2 | MI_3 🡨 PD_mean | 5.552 | 1.884 | .003 | EP_mean 🡨 MI_3 | .497 | .235 | .034 | GE_mean 🡨 MI_3 | 1.663 | .459 | .000 | 193.564 / |
|  | Constant | -1.950 | .895 | .029 | Constant | 3.488 | .205 | .000 | Constant | 7.542 | .400 | .000 | 205.774 |
| 3 | MI_4 🡨 PD_mean | 2.447 | .856 | .004 | EP_mean 🡨 MI_4 | .614 | .206 | .003 | GE_mean 🡨 MI_4 | .759 | .461 | .099 | 235.082 / |
|  | Constant | -.847 | .526 | .107 | Constant | 3.485 | .173 | .000 | Constant | 8.280 | .384 | .000 | 247.969 |
| 4 | MI_5 🡨 PD_mean | 2.179 | 1.040 | .036 | EP_mean 🡨 MI_5 | .076 | .187 | .685 | GE_mean 🡨 MI_5 | .695 | .471 | .140 | 199.061 / |
|  | Constant | -1.172 | .704 | .096 | Constant | 3.956 | .143 | .000 | Constant | 8.286 | .365 | .000 | 210.533 |
| 5 | MI_6 🡨 PD_mean | .719 | .741 | .332 | EP_mean 🡨 MI_6 | -.098 | .210 | .642 | GE_mean 🡨 MI_6 | .205 | .452 | .651 | 251.933 / |
|  | Constant | -.288 | .490 | .556 | Constant | 3.984 | .157 | .000 | Constant | 8.647 | .342 | .000 | 264.601 |
| 6 | MI_7 🡨 PD_mean | 1.098 | .757 | .147 | EP_mean 🡨 MI_7 | .092 | .222 | .680 | GE_mean 🡨 MI_7 | .407 | .478 | .394 | 246.634 / |
|  | Constant | -.207 | .488 | .672 | Constant | 3.869 | .181 | .000 | Constant | 8.485 | .396 | .000 | 259.302 |
| 7 | MI_8 🡨 PD_mean | 1.181 | .928 | .203 | EP_mean 🡨 MI_8 | .421 | .198 | .033 | GE_mean 🡨 MI_8 | 1.552 | .505 | .002 | 181.840 / |
|  | Constant | .080 | .573 | .889 | Constant | 3.612 | .174 | .000 | Constant | 7.446 | .442 | .000 | 193.312 |
| 8 | MI_9 🡨 PD_mean | .760 | .797 | .340 | EP_mean 🡨 MI_9 | -.184 | .250 | .462 | GE_mean 🡨 MI_9 | .561 | .519 | .280 | 244.942 / |
|  | Constant | .332 | .520 | .524 | Constant | 4.107 | .222 | .000 | Constant | 8.382 | .458 | .000 | 257.830 |
| 9 | MI_10 🡨 PD_mean | .045 | .857 | .959 | EP_mean 🡨 MI_10 | -.267 | .173 | .123 | GE_mean 🡨 MI_10 | .334 | .494 | .499 | 195.427 / |
|  | Constant | .140 | .580 | .809 | Constant | 4.110 | .130 | .000 | Constant | 8.504 | .367 | .000 | 206.637 |
| 10 | MI_11 🡨 PD_mean | 2.111 | 1.003 | .035 | EP_mean 🡨 MI_11 | -.101 | .228 | .656 | GE_mean 🡨 MI_11 | .061 | .626 | .923 | 203.048 / |
|  | Constant | -.401 | .588 | .496 | Constant | 4.041 | .202 | .000 | Constant | 8.646 | .563 | .000 | 214.774 |
| 11 | MI_12 🡨 PD_mean | -.345 | .870 | .692 | EP_mean 🡨 MI_12 | -.203 | .199 | .306 | GE_mean 🡨 MI_12 | -.013 | .477 | .979 | 210.533 / |
|  | Constant | .339 | .594 | .568 | Constant | 4.117 | .147 | .000 | Constant | 8.691 | .349 | .000 | 222.004 |
| 12 | MI_13 🡨 PD_mean | 1.320 | .894 | .140 | EP_mean 🡨 MI_13 | .064 | .211 | .763 | GE_mean 🡨 MI_13 | 1.038 | .499 | .037 | 223.867 / |
|  | Constant | -.157 | .602 | .794 | Constant | 3.984 | .183 | .000 | Constant | 8.000 | .436 | .000 | 236.535 |

*Note*. AIC = Akaike's information criterion; BIC = Bayesian information criterion.
